# Designer photonic dynamics by using non-uniform electron temperature distribution for on-demand all-optical switching times

**DOI:** 10.1038/s41467-019-10840-7

**Published:** 2019-07-04

**Authors:** Luke H. Nicholls, Tomasz Stefaniuk, Mazhar E. Nasir, Francisco J. Rodríguez-Fortuño, Gregory A. Wurtz, Anatoly V. Zayats

**Affiliations:** 1 Department of Physics and London Centre for Nanotechnology, King’s College London, London, WC2R 2LS UK; 2 Department of Physics, University of Warsaw, 00-927 Warsaw, Poland; 3 Department of Physics, University of North Florida, Jacksonville, FL 32224 USA

**Keywords:** Nanoscience and technology, Optics and photonics, Nanophotonics and plasmonics, Nonlinear optics, Physics

## Abstract

While free electrons in metals respond to ultrafast excitation with refractive index changes on femtosecond time scales, typical relaxation mechanisms occur over several picoseconds, governed by electron-phonon energy exchange rates. Here, we propose tailoring these intrinsic rates by engineering a non-uniform electron temperature distribution through nanostructuring, thus, introducing an additional electron temperature relaxation channel. We experimentally demonstrate a sub-300 fs switching time due to the wavelength dependence of the induced hot electron distribution in the nanostructure. The speed of switching is determined by the rate of redistribution of the inhomogeneous electron temperature and not just the rate of heat exchange between electrons and phonons. This effect depends on both the spatial overlap between control and signal fields in the metamaterial and hot-electron diffusion effects. Thus, switching rates can be controlled in nanostructured systems by designing geometrical parameters and selecting wavelengths, which determine the control and signal mode distributions.

## Abstract (web summary)

Here, the authors engineer a non-uniform electron temperature distribution through nanostructuring and demonstrate a sub-300 fs switching time. This can assist in the design of nanostructures for nonlinear optics, hot carrier extraction and photocatalysis

## Introduction

In laser science, optical signal processing and ultrafast nonlinear optics, the ability to control light with light at low powers and high speeds is a necessary evolutionary step towards integrated nonlinear photonic devices^1–3^. The use of nonlinear processes, stemming from free-carrier dynamics in good conductors, can provide some of the fastest and strongest Kerr-type nonlinear response^4,5^. This nonlinear response can be further augmented by nanostructure engineering, to enhance light-matter interaction and achieve favourable deviation from a general tendency of decreasing nonlinearity magnitude *χ*^(3)^ with increasing achievable switching rate *ν*: *χ*^(3)^*ν*~10^−9^ m^2^V^−2^s^−1^ ^6,7^. The inter- and intraband transitions in gold, induced by illumination with short-pulse-duration control light, create energetic nonequilibrium (initially non-thermalised) electrons that thermalise over a few hundred fs to adopt a Fermi-Dirac distribution with a defined electron temperature^8–10^. The conduction electrons then cool at a rate determined by the electron-phonon coupling, with relaxation of these thermalised electrons occurring over a timescale of a few picoseconds. The control light induced increase in temperature of the conduction band electrons translates into changes in the permittivity of the plasmonic nanostructure, which, for example, can be probed by another optical pulse^11,12^. When this free-electron nonlinearity is utilised in a suitable material arrangement or a nanostructure, the optical response, such as intensity^1,2,12,13^ and polarisation state^14,15^ of transmitted/reflected signal pulses, are modified all-optically at time scales shorter than those from commercial electronic devices and other nonlinear materials. For such plasmonic materials and nanostructures, the transient nonlinear response is often limited to picosecond-range time scales due to the aforementioned inherent relaxation of the thermalised electron gas, which ultimately determines the rate of the permittivity changes of the material^11,12^.

In most transient optical property studies, the electron temperature distribution induced by the absorbed light is assumed to be homogeneous across the structure, simplifying the problem when looking to simulate these transient nonlinear effects^16^. This is a fair assumption when considering timescales >1 ps, as any initial inhomogeneity will have passed in the first few hundreds of fs, due to the high electron Fermi velocity with which electrons spatially redistributed in the nanostructure. However, when looking at shorter times, local electron temperature variations may become significant in affecting the dynamics of the medium. This becomes particularly important in nanostructured systems that can support modes with complex spatial field distributions. The nanostructure interacting with light absorbs and stores energy in hot-electrons at locations mapped to spatial distribution of those modes where the absorbed energy is highest. This energy distribution can then be associated with local nonlinear changes of material permittivity, with hot-electron diffusion ultimately driving the dynamics of the optical properties of the nanostructure at very short time scales. Meanwhile, the electron energy will also be transferred to phonons in the material; the process which is usually described by a two-temperature model of electrons and phonons^17^. These processes have been shown to depend on restricted geometries in microstructures due to the modifications of the electron heat flow^18^.

Here, we take advantage of the rich modal structure of a plasmonic nanorod metamaterial, which provides a choice of wavelength-dependent intensity distributions in the nanorods. This in turn provides an opportunity to achieve inhomogeneous electron temperature distributions that have allowed us to obtain nonlinear switching determined by the rate of electron temperature diffusion rather than relaxation time alone, as in previous works. The effect on temporal dynamics of changing the overlap between the signal light mode within the metamaterial structure and the control light induced temperature distribution is also investigated. The measured dynamics can be used to study and engineer the electron temperature distributions and inform the design of nanostructures for specific applications in nonlinear optics, hot carrier extraction and photocatalysis.

## Results

### All-optical switching state

**Fig. 1:**
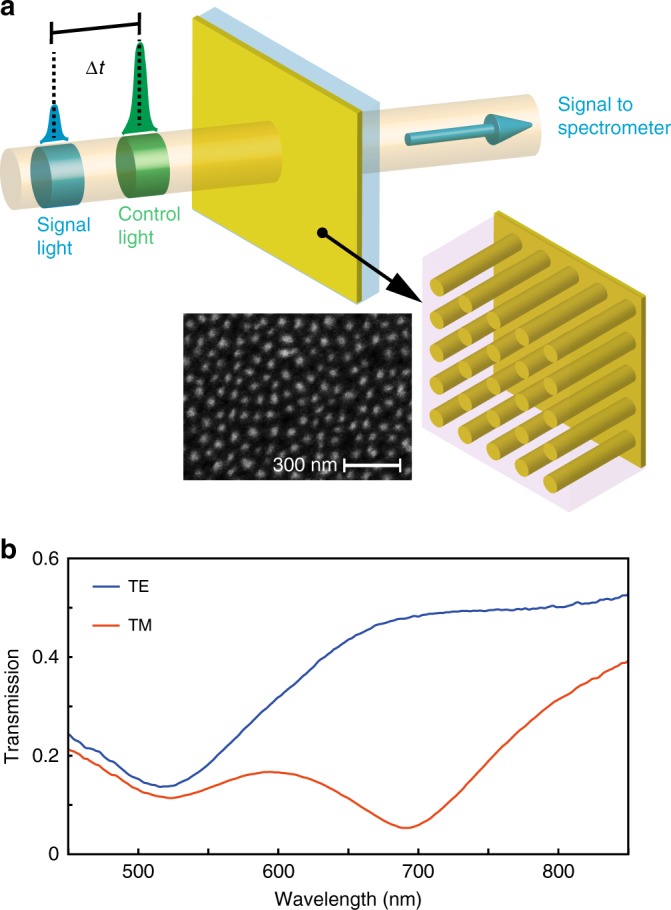
The nanorod metamaterial and its linear optical properties. **a** Schematic of the transient transmission measurements and the nanorod-based metamaterial. The control light is TE polarised, while the signal light is TM polarised. Inserts show the schematics and SEM image of the metamaterial. **b** Transmission spectra of the metamaterial (Au nanorods of approximate dimensions 400 nm length, 70 nm period and 30 nm diameter) for TE and TM polarised light at an angle of incidence of 45°

Transient spectroscopic measurements were carried out on an array of gold nanorods with length, diameter and period of 400 nm, 30 nm, and 70 nm, respectively, oriented perpendicular to a glass substrate and imbedded in an alumina matrix (Fig. 1a). This nanorod metamaterial is fabricated by an electrochemical process detailed in Methods. The nanorod metamaterial is optically uniaxial, due to the geometric anisotropy, and exhibits a resonance for extraordinary waves at around 700 nm wavelength (Fig. 1b), which corresponds to the spectral range of a transition from elliptic to hyperbolic dispersion^19,20^. The transition occurs where the permittivity tensor component along the nanorod axis changes its sign. Materials that exhibit such epsilon near zero (ENZ) characteristic have been shown to exhibit enhanced nonlinear optical effects^13,21–24^. These nonlinear effects can be used to affect the intensity and polarisation state of transmitted (signal) light, when illuminated with control light^14^. The designed ENZ region of the nanorod metamaterial structure has been shown to facilitate its high sensitivity sensing capabilities^22,25^, but also provides a method for controlling spontaneous emission and energy transfer between nearby emitters^26,27^.

To investigate the transient optical properties, a TE-polarised control light pulse of ~70 fs duration, at a central wavelength of 585 nm and peak power density of 16 GWcm^−2^ is incident on the metamaterial at an angle of 45° (Fig. 1a). This control light, which mainly excites intraband transitions in Au, being under the interband transition threshold energy of ~2.47 eV, changes the optical properties of the metamaterial by influencing the free-electron distribution in the conduction band of Au. The changes of the electron temperature can then be observed through modifications of the transmission spectrum of extraordinary waves using a low-intensity, broadband TM-polarised signal pulse (see Methods).

**Fig. 2:**
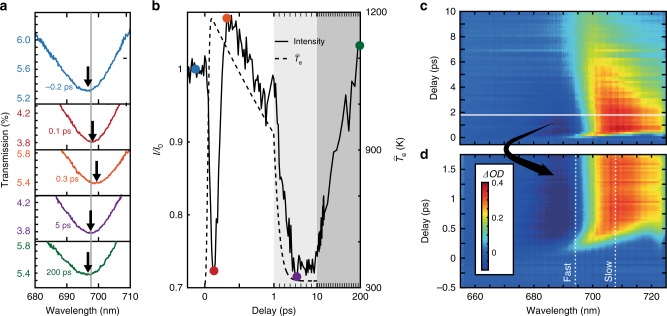
Transient optical properties of the nanorod metamaterial. Transient transmission spectra measured at a 45° angle of incidence through the nanorod metamaterial when illuminated with a 70-fs control light pulse, centred at a wavelength of 585 nm with a peak power density of 16 GWcm^−2^. **a** Transmission spectra of TM polarised signal light through the nanorod metamaterial measured for the different delay times. Arrows indicate the shifting resonance on control light illumination, with grey line indicating the monitored wavelength plotted in **b**. **b** Intensity (*I/I*_0_) of signal light with a wavelength of 697 nm at different delay times. Coloured markers indicate the times shown in **a** with the greyed regions indicating longer time scales (please note a changing *x*-axis scale). The dashed line is the simulated homogeneous temperature $\widehat {T_{\mathrm{e}}}$ of the electron gas after control pulse illumination. **c** The transient dynamics of the optical density (ΔOD) of the metamaterial from −0.5 to 10 ps. **d** High temporal resolution zoom of the sub-picosecond dynamics around the coincidence time of the control and signal pulse (below thin horizontal white line in **c**). Regions with fast and slow transmission variations are indicated with vertical white dashed lines

In the experiment, as the control pulse interacts with the metamaterial, the free-electron gas is excited and begins thermalising, leading to the increased electron temperature (sometimes known as a delayed nonlinearity^28^). This increase in electron temperature shifts the resonance to longer wavelengths. The red shift of this transmission minimum takes place during approximately the first 300 fs after the arrival of the control pulse (Fig. 2a). As the resonance approaches the monitored wavelength of 697 nm, the transmitted intensity experiences a fast drop of over 20%, reaching a minimum within ~100 fs. This minimum is obtained when the resonance is situated on top of the monitored wavelength (represented by the red curve and point in Fig. 2a, b). The transmission then rises back, as the resonance continues to move across and away from the monitored wavelength for an additional 200 fs, reaching transmission levels close to their original values (orange symbol and lines in Fig. 2a, b). The switching state duration, defined as the time that the transmission of the signal light takes to make a full cycle, i.e. ‘ON-OFF-ON’, due to the red shift of the resonance from one side of the monitored wavelength to the other, is 300 fs for the above discussion. The rate of the red shift of this resonance is measured to be ≈16 nm ps^−1^. At longer times, the intensity changes are driven by the relaxation of the temperature of the electron gas back to the ground state, resulting in a much slower blue shift of the resonance. The resonance moves back across the monitored wavelength, ~5 ps after control light excitation, and finally relaxes back to the ground state after ~200 ps (purple and green symbols and lines in Fig. 2a, b, respectively). The pronounced changes of the optical density (*ΔOD*) are also observed in a relatively broad spectral range around the metamaterial resonance (Fig. 2c, d). Spectral ranges with larger positive changes in optical density, of over 0.2, have longer switching state durations of up to 500 fs. At wavelengths above 705 nm, no such fast switching state is observed. The optical density only increases and then relaxes to the ground state over picoseconds timescales, as the red-shifting resonance stops short of crossing these wavelengths.

**Fig. 3:**
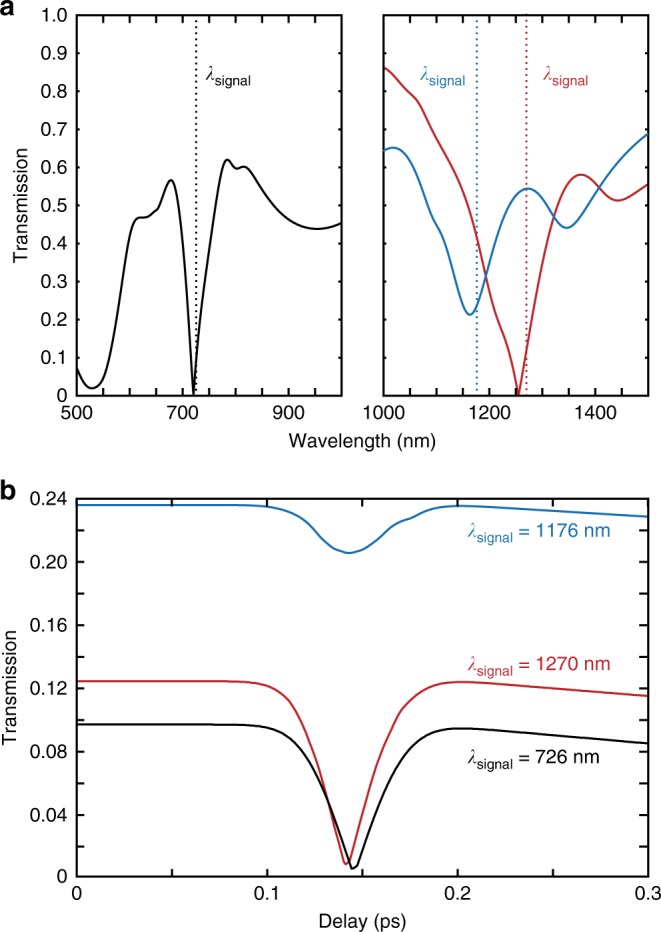
Transmission resonances and the switching time. **a** Simulated transmission spectra for the extraordinary wave at an angle of incidence of 30° for three metamaterials: (black) nanostructure parameters as in Figs. 1 and 2 but using bulk Au permittivity^42^ and two metamaterials optimised for higher transmission formed by nanorods with height, diameter, and period of (red) 680 nm, 25 nm, and 110 nm, respectively, and (blue) 550 nm, 27 nm, and 110 nm, respectively, for bulk Au permittivity. Dotted lines indicate the signal wavelength monitored in **b**. **b** Transient transmission of the metamaterials shown in **a** at the indicated signal wavelengths under 70 fs excitation changing the electron temperature to 1500 K

The switching time can be further decreased if the quality factor of the resonance is improved. This can be achieved with enhanced Au quality of the nanorods, replacing Au with lower-loss plasmonic material or designing more complex structures with higher-*Q* resonances. Working at the longer wavelength range^29^ where losses are also lower, the transmission resonance shift can be up to 40 nm for the same excitation conditions as in the experiment (red curve Fig. 3). A change in transmission of over 90% can be seen with the switching occurring within 80 fs. Interestingly, while the absolute transmission values change for different signal wavelengths, the switching time remains approximately the same: 80 fs for 726 nm and 1270 nm and 100 fs for 1176 nm operating wavelength). Like the measurements presented in Fig. 2, this fast switching is observed for a narrow range of wavelengths around the resonance, while outside this range slower switching is observed with higher absolute transmission changes.

In the model of a homogeneous increase of the electron temperature in the nanorods (see Methods), the simulations show significant disagreement between the rise time of this electron temperature and the time during which the resonance red shifts (Fig. 2b). Although the homogeneous electron temperature $\widehat {T_{\mathrm{e}}}$ has reached a maximum within 100 fs, the maximum red shift of the resonance has yet to be achieved. The maximum red shift is observed at around 300 fs, ~200 fs after the maximum average electron temperature has been reached. This indicates the importance of considering local heating of electrons, producing a non-uniform distribution along a nanorod, when describing the optical dynamics. In the following, we demonstrate that this discrepancy is determined by the rate of inhomogeneous hot electron dynamics (electron temperature diffusion) in the structure and the relative spatial overlap between the hot-electron distribution and the signal light mode. In the case when the signal and control light field distributions completely overlap in the nanostructure, the switching rate is ultimately determined by the electron-phonon relaxation, intrinsic to the material properties. Thus, the tailoring of control and signal light modes allows the tuneability of nonlinear dynamics beyond the inherent material properties.

### Inhomogeneous electron temperature

As previously mentioned, when looking at the dynamics at very short time scales, typically <1 ps, the electron temperature distribution generated by the mode profile of the control light in the nanostructure may become more influential on the optical dynamics. Therefore, to model the switching process described in the previous section, initial electron temperature distributions and their evolution should be considered. In the following considerations, the effect of non-thermalised excited electrons on the permittivity is not considered, as they are found to have short propagation lengths and <100 fs lifetimes^30–32^.

**Fig. 4:**
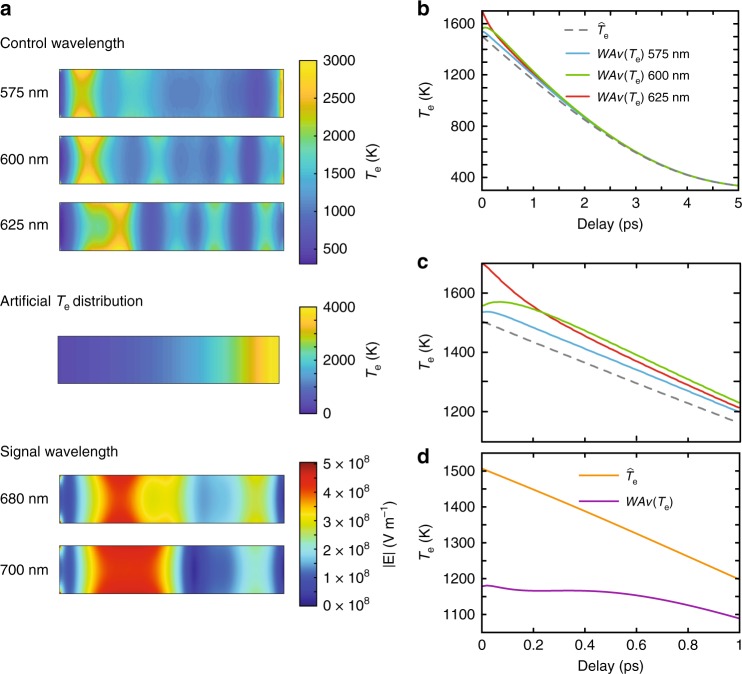
Evolution of electron temperature distribution in the nanorod metamaterial. **a** Cross sections of the electron temperature distributions in a unit cell nanorod of length, diameter, and period of 400 nm, 30 nm, and 70 nm, respectively. The distribution is induced by simulating control and signal light parameters as in Fig. 2 but with varied wavelengths. The electric fields produced in the unit cell structure by 680 nm and 700 nm signal light wavelengths are also shown to visualise the changing overlap between it and the electron temperature distributions. **b** Simulated electron temperature dynamics for different control wavelengths. The initial volume-average temperature is set to be 1500 K for each temperature distribution generated by the various pump wavelengths. *WAν*(*T*_e_) is then calculated by considering the overlap between the electron temperature distribution and the 680 nm signal mode in **a**. This is also compared to the homogeneous temperature model $\widehat {T_{\mathrm{e}}}$. **c** As **b** during the first ps. **d** Electron temperature dynamics of the artificial temperature distribution with a 680-nm signal light probe

The initial spatial distribution of the electron temperature is formed by the absorbed control light and determined by the wavelength-dependent field distribution inside the metamaterial (Fig. 4a, see Methods for the details of the simulations). This hot-electron distribution is then left to dissipate throughout the electron gas by diffusion in the nanorods and couple to phonons. The spatial overlap between the electron temperature distribution and the mode distribution supported by the metamaterial at the signal light wavelengths affects the transient response, especially at short times. Conversely homogeneous temperature considerations would provide the same transient response at all signal light wavelengths for the same energy absorbed in the nanostructure.

To illustrate this, the weighted average temperature was calculated (*WAν*(*T*_e_)), which is the average temperature of each discrete cell element in the rod weighted by the signal light intensity in that cell (Fig. 4b, see Methods). The dynamics of *WAν*(*T*_e_) for different initial temperature distributions induced by three different control light wavelengths can now be compared with the dynamics observed when assuming a uniform temperature across the nanorod. In all cases, the initial spatially averaged electron temperature over the rod is 1500 K. The overall dynamics observed over a 5-ps time span is similar for all initial distributions (Fig. 4b). However, when analysing the first picosecond (Fig. 4c), large deviations from the homogeneous temperature consideration are evident, related to the inhomogeneous temperature distributions. For example, for a signal wavelength of 680 nm and a control wavelength of 625 nm, the starting *WAν*(*T*_e_) is 1700 K, much higher than the homogeneous temperature, as well as the *WAν*(*T*_e_) achievable with the other control wavelengths. The relaxation is also monotonic in this case, whereas *WAν*(*T*_e_) for control wavelengths of 575 nm and 600 nm peak at 50 fs and 100 fs, respectively, after the initial distribution starts to relax. The maximum local temperature in the rod for the three control wavelengths 575, 600, and 625 nm are ~40, 50, and 32 kK, respectively, in the parts of the nanorod closer to the surface (not visualised in the central cross-section shown in Fig. 4a). These temperatures are much higher than the volume average but are situated in a small volume of the rod within the mode ‘hot spots’. With proper mode design, high efficiency conversion of control light intensity to maximum local electron temperature can be achieved. This would be beneficial where only small volumes of the material permittivity need to be changed rather than the bulk properties of the structure, for example for the extraction of hot electrons in order to induce photocatalytic processes^32–35^.

**Fig. 5:**
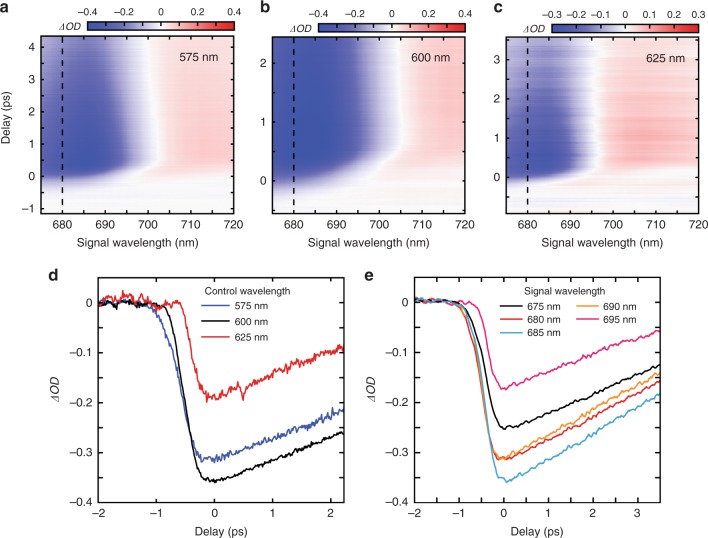
Effect of changing control and signal light wavelength on transient optical properties. Transient dynamics spectra for different control light wavelengths of **a** 575 nm, **b** 600 nm and **c** 625 nm. The sample is illuminated at an angle of 45° using a ×4 objective. The peak power density for each pump wavelength was set around 10 GWcm^−2^ with pulse duration around 70 fs for each wavelength. **d** Measured *ΔOD* at a signal wavelength of 680 nm, indicated by the dashed line in **a**–**c**, for different control wavelengths. **e** Measured *ΔOD* at different signal wavelengths for a control wavelength of 575 nm. In **e** and **d**, zero delay is taken to be the point at which the value of |*ΔOD*| is maximum

The effect of the inhomogeneous temperature was verified using transient *ΔOD* measurements for three control light wavelengths of the same peak power density (Fig. 5). Each control wavelength produces very different transient maps (Fig. 5a–c), with strongest shift and amplitude change seen for 600 nm control light wavelength and weakest for 625 nm. The effect of changing control light wavelength on the excitation dynamics is markedly seen (Fig. 5d). The fastest measured transmission change occurs for the 625 nm distribution. This agrees qualitatively on comparison with the simulations in Fig. 4 as it has the electron temperature distribution with the strongest overlap with the 680 nm signal light. The control light with wavelength of 575 nm and 600 nm induces approximately 100% and 50% longer switching time, respectively, than the 625 nm light. Different dynamics is also seen for a fixed control wavelength and a changing signal wavelength (Fig. 5e), with a signal wavelength of 695 nm showing a >30% shorter switching time than the other signal wavelengths shown.

To further demonstrate the potential strength of our approach for engineering nonlinear dynamics, Fig. 4d shows the simulated temporal response of *WAν*(*T*_e_) using a temperature distribution designed with very low spatial overlap with the signal light mode, which can be produced by using wavelengths that excite mainly interband transitions, i.e., *λ* < 500 nm. In this case, the *WAν*(*T*_e_) is suppressed considerably to below 1200 K, with very little change over the first 600 fs.

### Varying angle of incidence

**Fig. 6:**
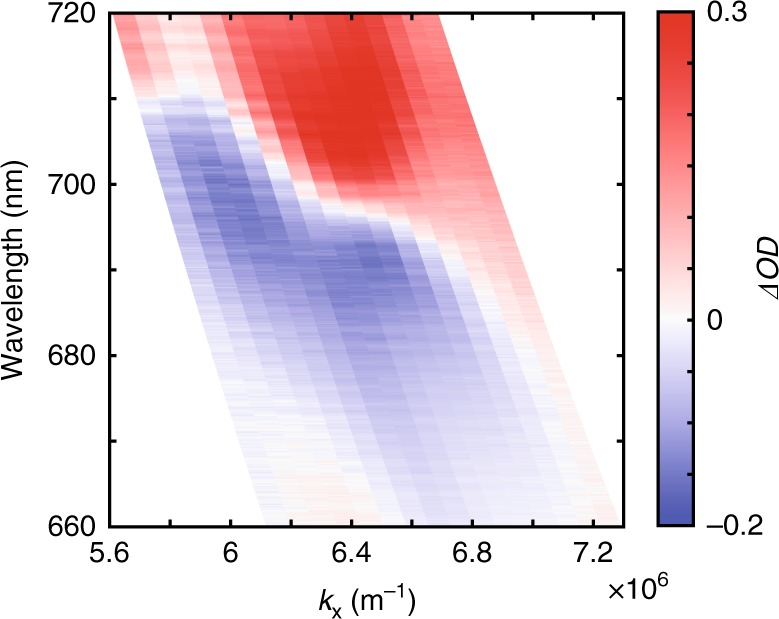
Changing dynamic response with angle of incidence of  control light. The variations of optical density (*ΔOD*) at a time of 1 ps after the control pulse for different angles of incidence of the signal light (the wavector perpendicular to the nanorod axis, $k_x = k_0{\mathrm{sin}}\theta$ with $k_0$ being the incident wavevector in air and $\theta$ being the angle of incidence)

Additional to changing the wavelength of the signal mode, another way of affecting the induced electron temperature distribution is to vary the angle of incidence of the signal and/or control light. The nonlinear response of the metamaterial will then be modified due to the changing spatial distribution of the field enhancement with angle of incidence. In contrast to a plane wave illumination at a fixed angle of incidence discussed above, when focussing the control light, a range of incident angles is generated. In the experiment, this range of angles is estimated to be ±6° around the 45° central angle of incidence of the illuminating objective with NA = 0.10. From the measured dynamic dispersion of the metamaterial around the resonant wavelength (near ENZ wavelength) and at a time of 1 ps after the control light pulse, we can clearly see that the amplitude of the nonlinear change in optical density is strongly incident angle dependent (Fig. 6). This effect is also evident at shorter time scales, down to around 300 fs. Furthermore, the sign of the nonlinear change, i.e. supressed or increased transmission, also depends on the incident angle. A wavelength range exists, at ~690–710 nm, whereby the increase of angle of incidence results in the *ΔOD* at a given time changing sign from negative to positive. These effects can be attributed to the strong nonlocal effects present in the nanorod metamaterial^13,36^, and also the change in the field overlap between signal and control light when altering incident angle, even when the wavelengths are fixed. This provides another way of tailoring the dynamic response, where incident angle can affect strength and sign of the nonlinear response of the metamaterial.

## Discussion

We have shown the importance of considering inhomogeneous electron temperature distributions, when dynamics at very short time scales are of interest. An ultrafast switching state has been demonstrated with tuneable duration of 200–500 fs, dependent on the mode overlap of the signal and control light modes. The influence of mode overlap on the optical dynamics was demonstrated both through experiment and numerical modelling by changing angle of incidence and altering the control and signal light wavelengths. The sign and amplitude of the change of the transmission through the structure can also be altered through the same parameters. The dynamic effect is shown to be due to the finite time that the inhomogeneous electron temperature takes to diffuse through the anisotropic plasmonic structure. It is shown that after 1 ps electron temperature diffusion has rendered the electron temperature distributions homogeneous. The time required to eliminate electron temperature gradients can be confirmed by simplistic estimates, using the Fermi velocity of electrons in Au and the nanorod length, to be ~300 fs, which is consistent with the experimental observations and modelling presented above.

The results show that vastly different optical environments can be created within the same nanostructure, as local electron temperature in ‘hot-spots’ were found to be an order of magnitude larger than the average temperature. By designing geometries that can fully exploit these temperature-diffusion-based nonlinear dynamics, the temporal control of the local permittivity in the nanostructure can be achieved. This permittivity control is driven by the free-electron response in plasmonic metals. However, materials that exhibit efficient optical Kerr effect, which depend on local temperature, could also be utilised. Semiconductor nanostructures are one suitable material if the excitation of free carriers is responsible for the nonlinearity.

The active control of the local permittivity at the nanoscale could provide novel functionality not only for Kerr-nonlinearity tailoring, but also for controlling spontaneous emission, influencing both energy transfer and decay rates, second-harmonic generation^37,38^ and sensing applications for dynamic control of refractive index sensitivity. Applications for this local design of electromagnetic environment include future display technologies, nanoscale light sources, and super resolution imaging. Furthermore, on implementing this method of managing dynamic response, there lies new potential to increase the versatility of active all-optical devices by producing both slow and fast optical modulation and switching with the same nanophotonic environment. This can be achieved by spatial design of the electron temperature through the generation of distinct control light modes within the nanostructure.

## Methods

### Nanorod fabrication

The nanorod metamaterial consists of a periodic array of metallic nanorods oriented perpendicular to a 1-mm-thick glass substrate and embedded in an alumina matrix. The fabrication is carried out by a self-assembly process, which leads to large area homogeneous arrays. The process starts with a meticulously clean glass substrate to which an adhesion layer of 20 nm of Tantalum Pentoxide (Ta_2_O_5_) is deposited by physical vapour deposition. Similarly, an under layer of gold is added with thickness approximately 7 nm. A layer of aluminium is then deposited such that on anodization of the sample the aluminium layer forms a 400-nm thick layer of porous alumina (Al_2_O_3_). The anodization conditions are tuned such that the formed pores have a period of 70 nm and a diameter of 30 nm into which gold can be electro-deposited to form the completed metamaterial sample.

### Transient optical measurements

Transient transmission measurements were achieved in a standard colinear pump-probe setup geometry. In principle, the experiment need not be restricted to a colinear setup. Indeed, differing angles of incidence of control and signal light may provide an extra degree of freedom when designing electron temperature distributions and overlap between signal and control modes within the nanostructure, as well as illumination from the opposite sides of the sample. Moreover, the effect can be seen in both transmission and reflection, although the resonances will be in different spectral positions. The experiment uses two amplified outputs of a Ti:sapphire (Coherent-Micra) femtosecond laser system, both at a central wavelength of 800 nm. The control pulse is generated by one of these outputs that is sent to an optical parametric amplifier, allowing tuneable wavelengths from 400 to 1200 nm with a pulse width of around 50 fs. Accounting for dispersive optics in the system the pulse incident on the nanostructure has duration of around 70 fs. The other output from the Ti:sapphire laser, used as the signal pulse in the experiment, is passed through a sapphire plate in order to generate a white light spectrum and delayed in time from the control pulse, via an optical delay line, in order to probe the transient optical properties of the metamaterial sample. Both pulses are focussed onto the sample at an angle of 45° using a ×4 objective and collected in transmission by a ×50 objective. The signal pulse is directed to a spectrometer for full spectral analysis. The detection optics are designed such that the addition of a lens allows imaging of the Fourier plane, to gain not only spectral but k-vector information, used to measure the dynamic dispersion of the sample.

### Modelling inhomogeneous electron temperature

At short time scales, the local temperature of the electrons across the nanostructure becomes important. The two-temperature model (TTM) is used to describe the evolution of the thermalized conduction electrons in gold, which can be modelled as a gas with a certain temperature. In the pump-probe experiment, the energy of the pulse causes heating of the conduction band electrons. This heat is then dissipated in the metal first by electron temperature diffusion, then electron-phonon interactions, which allows heat to be lost from the electron gas to lattice vibrations and finally phonon-phonon interactions^39,40^. A further assumption of this model is that thermalisation of the electron gas occurs instantaneously, that is to say a Fermi-Dirac distribution can always be used to describe the gas. Given these assumptions the temperature dynamics of the system can be described by the following set of coupled equations^40^:

1 $$C_{\mathrm{e}}\left( {T_{\mathrm{e}}} \right)\frac{{\partial T_{\mathrm{e}}}}{{\partial t}} = \mathop{\nabla }\limits^{\rightharpoonup} \left( {k_{\mathrm{e}}\left( {T_{\mathrm{e}}} \right)\mathop{\nabla }\limits^{\rightharpoonup} T_{\mathrm{e}}} \right) - G\left( {T_{\mathrm{e}} - T_{\mathrm{p}}} \right) + Q$$

2 $$C_{\mathrm{p}}\frac{{\partial T_{\mathrm{p}}}}{{\partial t}} = G\left( {T_{\mathrm{e}} - T_{\mathrm{p}}} \right)$$

where *C*_e_ = 67.7 J m^−3^ K^−3^ × *T*_e_ and *C*_p_ = 3.5 J m^−3^ K^−2^ are the heat capacities of the electrons and phonons, respectively, *T*_e_(x,y,z) and *T*_p_(*x,y,z*) are their local temperatures, $k_{\mathrm{e}} = k_{{\mathrm{e}}0}\frac{{T_{\mathrm{e}}}}{{T_{\mathrm{p}}}}$ is the electron diffusion coefficient with *k*_e0_ = 318 W m^−1^ K^−1^ and *Q*(*x,y.z*) is the energy dissipated locally in the nanorod^41^. When considering homogeneous energy dissipation, *Q* is taken spatially independent, causing a homogeneous temperature distribution. In this case, Eq. 1 is greatly simplified as the gradient term is equal to zero.

In this study, however, we consider inhomogeneous temperature distributions. Therefore, the gradient term in Eq. 1 is nonvanishing as it deals with the spatial distribution of the electron temperature. Local electron temperature must be calculated using the modal distribution of the pump beam and the power it dissipates at each point in the nanorod^16^. This is done by splitting the rod into mesh elements and calculating the power dissipated in each, *Q*(*x,y,z*). Once the initial electron temperature distribution is calculated, Eqs. 1 and 2 can then be implemented locally in each element of the rod, considering the heat transfer between adjacent elements. At the boundary of the rod the temperature gradient is set to zero to avoid erroneous electron transfer out of the nanorod bath. Large changes in local temperature occur, so very short time steps of at most 0.01 fs must be taken for the solution to converge. A weighted average electron temperature *WAν*(*T*_e_) can then be calculated by considering the overlap between the signal light mode in the metamaterial and the temperature distribution at each time step.

For comparison, the homogeneous electron temperature $\widehat {T_{\mathrm{e}}}$ is calculated as the average temperature over the rod volume:

3 $$\widehat {T_{\mathrm{e}}} = \frac{1}{V}\mathop {\smallint} \nolimits T_{\mathrm{e}}\left( {x,y,z,t = 0\,s} \right){\mathrm{d}}V$$

where, *T*_e_(*x,y,z,t* = 0 *s*) is the temperature distribution induced by the control pulse at a time *t* = 0* s* and *V* is the volume of the rod. The intensity of each control wavelength is set such that value of this volume integral is equal in all cases. This temperature is then taken as the homogeneous temperature across the rod and allowed to relax according to Eqs. 1 and 2.

When considering the inhomogeneous temperature distribution, the interaction between the spatial temperature distribution and the probe signal mode is taken into account in the quantity *WAν*(*T*_e_). This is calculated at different delay times *t*_0_

4 $${\mathit{WA\nu}}\left( {T_{\mathrm{e}},t = t_0} \right) = \frac{{{\int} {\left| {{\mathbf{E}}_{\mathrm{s}}\left( {x,y,z} \right)} \right|^2T_{\mathrm{e}}\left( {x,y,z,t = t_0} \right){\mathrm{d}}V} }}{{{\int} {\left| {{\mathbf{E}}_{\mathrm{s}}\left( {x,y,z} \right)} \right|^2{\mathrm{d}}V} }}$$

where, **E**_s_ is the electric field of the signal mode. *WAν*(*T*_e_) then gives a value that is the average temperature weighted by the probing signal mode.

## Data Availability

The data that support the findings of this study are available from the corresponding author upon reasonable request.
